# Impaired dendritic cell proinflammatory cytokine production in psoriatic arthritis

**DOI:** 10.1186/1479-5876-9-S2-P58

**Published:** 2011-11-23

**Authors:** Mark H Wenink, Kim CM Santegoets, John Butcher, Lenny van Bon, Femke GM Lamers-Karnebeek, Wim B van den Berg, Piet LCM van Riel, Iain B McInnes, Timothy RDJ Radstake

**Affiliations:** 1 Dept. of Rheumatology, Nijmegen Center of Infection, Inflammation and Immunity (N4i) and Nijmegen Centre for Molecular Life Science (NCMLS), Radboud University Nijmegen Medical Centre, Nijmegen, The Netherlands; 2 Division of Immunology, Infection and Inflammation, Glasgow Biomedical Research Centre, University of Glasgow, Glasgow, Scotland, UK

## Background

The pathogenesis of Psoriatic Arthritis (PsA) remains poorly understood. The underlying chronic inflammatory immune response is thought to be triggered by unknown environmental factors potentially arising from a defective immune function.

## Aim

To determine whether an impaired acute inflammatory response by Dendritic Cells (DCs) might compromise the clearance of bacteria and predispose to chronic inflammation in PsA.

## Patients and methods

The cytokine production by DCs from 13 healthy controls, 11 Rheumatoid Arthritis, 13 PsA and 8 psoriasis patients from The Netherlands and from 6 Scottish healthy controls and 6 PsA patients towards *Mycobacterium tuberculosis*, *Mycobacterium avium paratuberculosis* and a range of other bacteria and TLR ligands was determined. Phenotypical differences involved in cellular responses against (myco)bacteria were determined by qPCR and flow cytometry.

## Results

The secretion of the proinflammatory cytokines TNFα and IL-6 by PsA DCs was impaired upon in vitro challenge with mycobacteria and TLR2 ligands compared to DCs from healthy controls and RA patients. DCs from psoriasis patients did not demonstrate such a dampened reaction towards mycobacteria. This impairment demonstrated by PsA DCs was associated with elevated serum CRP levels. The expression of TLR2 and other receptors known to mediate mycobacterial recognition was unaltered. The intracellular TLR(2) inhibitors SOCS3 and A20 were more highly expressed in DCs from PsA patients. PsA DCs further demonstrated up regulated levels of the autophagy-related genes ATG16L1, NOX2 and LL37; molecules implicated in the immune response against intracellular bacteria.

## Conclusion

Our findings indicate that DCs from PsA patients have a disordered immune response towards some species of (myco)bacteria. This might predicate to impaired immune responses to, and in turn impaired clearance of, these bacteria, setting the stage for the chronic inflammation of joints, entheses, skin and the gut found in PsA.

